# Coupling effects of human serum albumin and sodium chloride on biological desiccation patterns

**DOI:** 10.1016/j.heliyon.2023.e19970

**Published:** 2023-09-07

**Authors:** Jihong Wang, Min Zhang, Jun Wang, Ruoyang Chen

**Affiliations:** a School of Physics and School of Materials Science and Engineering, East China University of Science and Technology, Shanghai, 200237, China; b Wenzhou Institute, University of Chinese Academy of Sciences, Zhejiang, 325000, China

**Keywords:** Desiccation, Cracking pattern, Crystal pattern, Bio-fluid

## Abstract

Desiccation patterns of plasma sessile drops have attracted increasing attention, not only because of the fantastic underlying physics, but also due to their potential of being health diagnostic tools. However, plasma is a multicomponent system, which contains macromolecular proteins and inorganic salts; these components have complicated interactions to define pattern morphologies. Unfortunately, mechanisms of coupling effects of main components on pattern morphologies are still not clear, thus limiting their diagnostic applications. Here we show the coupling effects of human serum albumin (HSA) and sodium chloride (NaCl) on plasma desiccation patterns. Our experiments indicate that NaCl enhances the “coffee ring” effect of HSA to promote its aggregation at the peripheral region and narrows down its aggregation area; this would influence the distribution of internal stresses, resulting in a larger number of radial cracks, with a larger width but a shorter length, than cracks in pure HSA. In the meantime, HSA experiences the gelation process that propagates from the peripheral region to central region and causes the spatiotemporal deviation in the degree of solidification, which induces a higher concentration of NaCl in the central region, thus leading to the formation of crystal patterns. Our further experiments demonstrate that these characteristic patterns are correlated to the variation in the concentration of NaCl, which can be caused by hyponatremia and hypernatremia in real biofluids. Our findings not only provide a new mechanistic insight into biological desiccation patterns, but also bridge the gap between the understanding and diagnostic applications of these desiccation patterns.

## 1 Introduction

Desiccation of a biological sessile drop on a smooth surface experiences complex physical processes, e.g., material redistribution induced by non-uniform distribution of evaporation rate above the drop, gelation caused by aggregation of macromolecular proteins, buildup/release of internal stress and salt crystallization [1,2]. After desiccation, some characteristic patterns, which are typically featured by cracking patterns and/or crystal patterns, are left on the surface [[1], [2], [3]]. The morphological characteristics of these patterns have recently been found to be a potential indicator for some diseases, e.g., anemia, hyperlipidemia, and neonatal jaundice [4,5]; these diseases induce the deviation of compositions, structures and functions of biological dispersions in biological fluids from healthy donors, which would influence the desiccation process of the sessile drop, and thus leading to distinguished desiccation patterns [6]. Therefore, biological desiccation patterns have been considered to be a low-cost and facile potential diagnostic tool to interpret health conditions of donors [[7], [8], [9]]. However, these potential applications are still limited by the insufficient understanding of mechanisms underlying the pattern formation [10,11].

To advance diagnostic applications of biological desiccation patterns, great efforts have been paid on the mechanistic understanding of pattern formation, particularly for human blood plasma, which has both characteristic features of cracking patterns and crystal patterns [[12], [13], [14], [15]]. The cracking patterns of plasma sessile drops, either in radial or orthoradial direction, have been reported to be correlated to the non-uniformly spatial and temporal distribution of local dominant stresses, which were induced by the completion between the continuous drop shrinkage and the adhesion of biological dispersions to the supporting substrate [16]. The crystal patterns, which were concentrated in the central region of plasma desiccation patterns, have been found to be caused by the heterogeneous distribution of macromolecular proteins and inorganic salts during drop drying [16]. Most of these conclusions have been drawn while taking the biological sessile drop as a whole [17,18]. However, biological fluids are complicated multicomponent systems. For example, human blood plasma is composed of macromolecular proteins, e.g., human serum albumin (HSA), and inorganic salts, e.g., sodium chloride (NaCl); these components would have coupling effects on the evaporation behaviors of the sessile drop and thus the morphologies of desiccation patterns.

Recently, numerous works have been carried out on coupling effects of macromolecular proteins and inorganic salts [[19], [20], [21], [22], [23], [24], [25], [26], [27]]. The study on binary mixtures of bovine serum albumin (BSA) and phosphate buffered saline (PBS) indicates that intermolecular interactions of BSA are dominant over the interactions between BSA and PBS in the peripheral region and *vise verse*, thus leading to the formation of regional desiccation patterns [22]. In another earlier work, coupling effects of BSA and PBS on cracking patterns have been correlated to the competition between the evaporation-induced and relaxation-induced stress [25]. On the other hand, the mixtures of BSA and chloride salts also become popular for mechanistic study. They can form crystal patterns with various morphologies, e.g., rosette, scallop, Chinese arrow and dendrite shapes [20]. These crystal patterns can be changed when the system contains micromolecular protein, e.g., lysozyme [23]. Furthermore, in the presence of proteins, the altering of initial ratio of two chloride salts leads to the transformation between a continuous dendritic structure and a snow-flake type structure [26]. Despite great efforts on the mechanistic study [[28], [[28]a]], [29], the coupling effects of HSA and NaCl on pattern morphologies are still not fully understood [29].

In this work, we aimed to investigate the coupling effects of HSA and NaCl on biological desiccation patterns via simplifying the human blood plasma into a bi-component system, in which the concentration of each component was same as the plasma in a healthy adult, i.e., the concentration of HSA and NaCl was controlled at 50 g/L and 8 g/L, respectively. We employed a scanning electron microscope (SEM), equipped with an energy-dispersive X-ray (EDX), to investigate microstructural morphologies and chemical compositions of desiccation patterns for the mixture of HSA and NaCl (HSA + NaCl), when comparing with pure HSA and NaCl. We disclosed the mechanisms underlying the coupling effects of HSA and NaCl on both cracking patterns and crystal patterns. Based on our mechanistic understandings, we correlated these characteristic patterns to the variation in the concentration of NaCl, which can be caused by hyponatremia and hypernatremia in real biofluids.

## 2 Experimental section

The glass cover slides (German BRESSER) were immersed in anhydrous ethanol for 2 h, and subsequently rinsed with ethanol, followed by the final wash using ultra-pure water (18 MΩ cm^−1^). In order to keep the concentration of each component at the same level as the plasma in a healthy adult, we prepared the mixture of HSA and NaCl in concentrations of 50.00 g/L and 8.19 g/L, respectively. A drop of HSA + NaCl, pure HSA or NaCl solution, with a volume of 2 μL, was carefully deposited onto the glass surface. The height of the drop addition was controlled at ∼0 mm to avoid the possible interference of drop evaporation caused by the inertial force. The sessile drop was left to dry in a controlled environment with a temperature of 30.0 ± 0.5 °C and a relative humidity of 55.0 ± 0.5%, respectively. After drying for 2 h, desiccation patterns can be obtained. To explore potential applications of desiccation patterns in health diagnosis, HSA samples with varying concentrations of NaCl (i.e., 4.09 g/L, 8.19 g/L, and 16.38 g/L) were prepared. The desiccation patterns of mixture drops were prepared in the same way as those mentioned above. We have repeated experiments at least five times and prepared six individual samples in each experiment.

For characterization of the morphological details and microstructure of different regions within desiccation patterns, a high-power microscope (Olympus, Japan) and a CountorGT-K 3D optical profilometer (Bruker, USA) were employed. In addition, an SU8010 SEM instrument (Hitachi, Japan) was used along with EDX analysis to obtain elemental information. The conditions for EDX analysis were set at 15 kV voltage and 10 μA current with face detection mode selected and the detection area limited to less than 1 μm^2^. Based on the morphological characterization, we performed the statistical analysis of the width of radial cracks, the number of radial cracks, the ratio of crack length to radius, the crystal length and crystal width of the main grain in desiccation patterns on these samples by using ImageJ and Origin software packages. The elemental analysis was performed by taking an average of the EDX results in six individual samples.

## 3 Results and discussion

### 3.1 Coupling effects of NaCl and HSA on cracking patterns

**Fig. 1:**
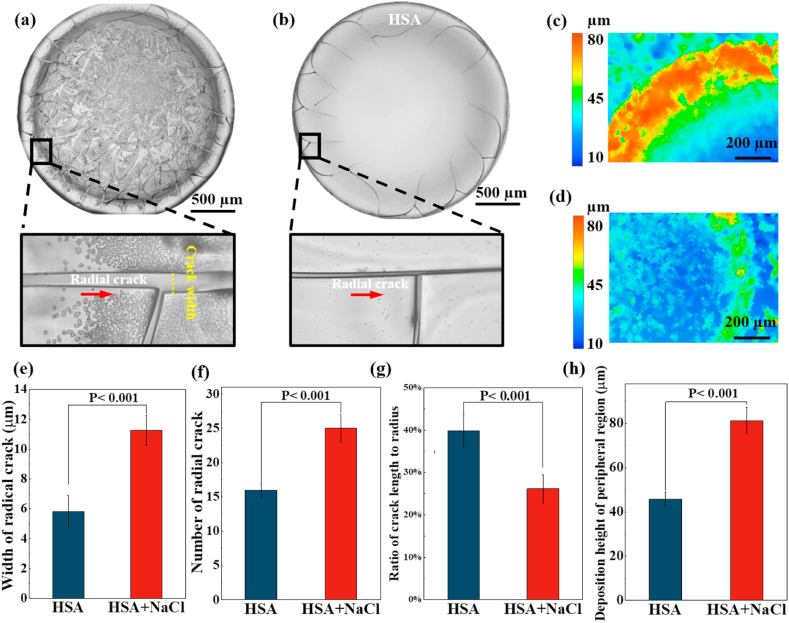
Desiccation patterns of (a) mixture of HSA and NaCl (HSA + NaCl) and (b) HSA; three-dimensional (3D) profiles of desiccation patterns of (c) HSA + NaCl and (d) HSA; (e) The width of radial cracks in desiccation patterns of HSA and HSA + NaCl; (f) the number of radial cracks in desiccation patterns of HSA and HSA + NaCl; (g) ratio of crack length to radius in desiccation patterns of HSA and HSA + NaCl; (h) deposition height of the peripheral region in desiccation patterns of HSA and HSA + NaCl.

The desiccation patterns of HSA + NaCl, as shown in Fig. 1a, are similar to plasma desiccation patterns, which had been reported in our previous work [18]. The patterns also have two distinguishable regions from the outer to inner, i.e., the peripheral region and the central region. The peripheral region is mainly composed of cracking patterns, in both of radial and orthoradial directions. The cracks in radial direction (radial cracks) have an ordered distance and extend from the peripheral region towards the central region. In the central region, there are numerous complex crystal patterns, which appear to be the tree-like structure, i.e., dendritic crystal.

We compared cracking patterns of HSA + NaCl with pure HSA (Fig. 1a and b) and found that both the number and the width of radial cracks of HSA + NaCl are larger than these of HSA, while the length of radial cracks of HSA + NaCl are less than that of HSA. Our quantitative analysis shows that the average width of radial cracks of HSA desiccation patterns is 5.8 μm, which is much less than that of HSA + NaCl of 11.2 μm (Fig. 1e). Moreover, the number of radial cracks of pure HSA is 16 ± 1, which is less than that of HSA + NaCl of 25 ± 2, as shown in Fig. 1f; this means that the addition of NaCl into HSA leads to the significant increase of both the width and number of radial cracks in desiccation patterns, while decreases the crack spacing. On the other hand, the normalized length of radial cracks experiences a considerable reduction from 40% to less than 26% (Fig. 1g). To further analyze the morphological differences in these desiccation patterns, we reconstructed the three-dimensional (3D) profile of desiccation patterns by using an optical profilometer. Clearly, the apex in the peripheral region of desiccation patterns of HSA + NaCl (Fig. 1c) is much higher than that of desiccation patterns of pure HSA (Fig. 1d); the deposition height in the peripheral region of desiccation patterns of HSA + NaCl is ∼81 μm, which is larger than that of pure HSA of ∼45 μm (Fig. 1h). This phenomenon suggests that the presence of NaCl promotes the “coffee ring” effect of HSA during evaporation, resulting in higher protein accumulation at the peripheral region.

### 3.2 Coupling effects of HSA and NaCl on crystal patterns

**Fig. 2:**
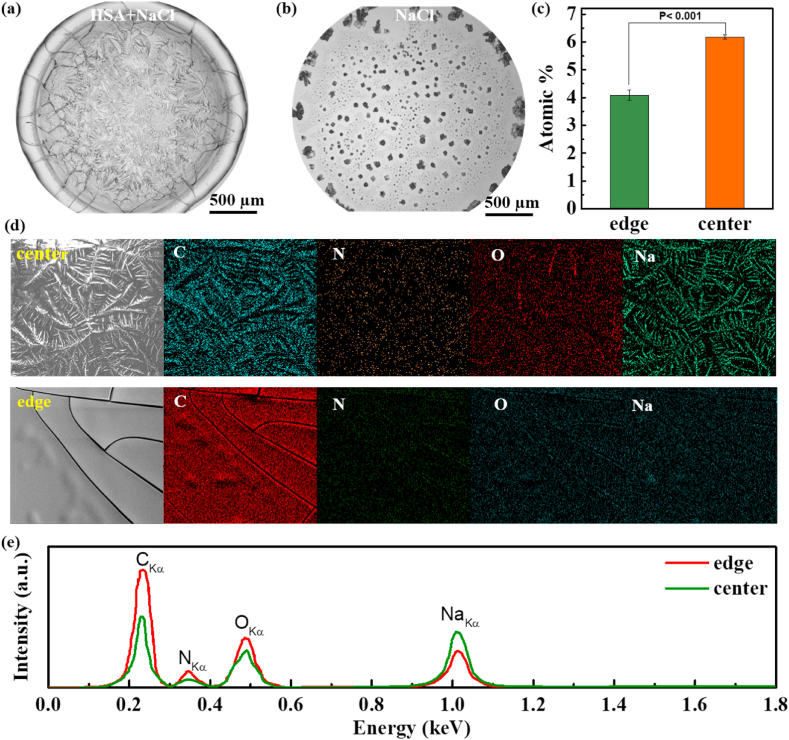
SEM images of desiccation patterns of (a) HSA + NaCl and (b) pure NaCl; (c) content of Na atoms in the central and peripheral regions; (d) EDX mappings of crystal patterns in the central region and cracking patterns in the peripheral region; (e) EDX analysis on different areas of HSA + NaCl desiccation patterns in (d).

The morphological details in different regions of desiccation patterns of HSA + NaCl were characterized by SEM, as shown in Fig. 2a. We note that the boundaries of crystal and radial cracking patterns coincide with those of adjacent patterns, and that these distinct patterns can be seamlessly integrated into a unified whole. It indicates that the crystallization process occurs prior to the cracking process. Additionally, EDX mapping images in Fig. 2d, EDX analysis in Fig. 2e and quantitative calculations in Fig. 2c could illustrate that crystal patterns are mainly composed of inorganic salt (i.e., NaCl); these results also indicate that the NaCl content in the central region is obviously higher than that in the peripheral region. In order to further investigate the influence of HSA on NaCl crystal patterns, the desiccation patterns of pure NaCl were introduced as a control (Fig. 2b). It exhibits a prominent manifestation of the “coffee ring” effect, wherein NaCl accumulates along the perimeter of the sessile drop, resulting in the formation of crystals. All these indicate that the presence of HSA inhibits the “coffee ring” effect of NaCl during drop drying. Interestingly, this phenomenon has also been found in some other simplified biological systems, e.g., mixtures of phosphate buffer saline (PBS) and bovine serum albumin (BSA)/lysozyme (Lys) [22]. These authors considered that the concentration of salts in the peripheral region of the sessile drop could approach to zero, while that in the central region could reach its maximum value [22].

### 3.3 Mechanism underlying the coupling effects

**Fig. 3:**
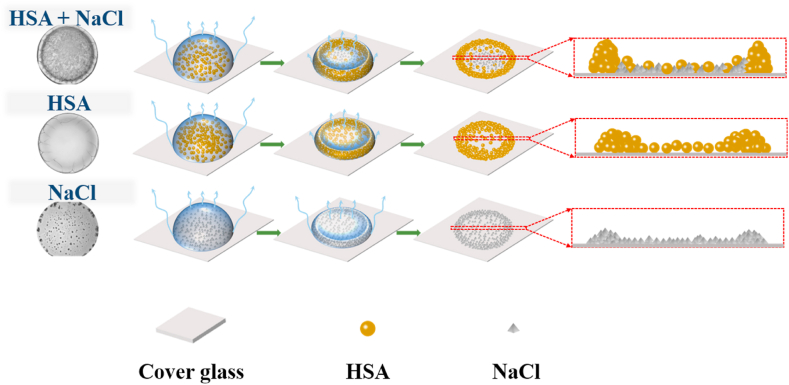
Schematics of drying process of sessile drops of HSA + NaCl, pure HSA and NaCl drops.

We first discuss the physics of the formation of cracking patterns in the peripheral region. The presence of NaCl significantly enhances the “coffee ring” effect of HSA and promote its accumulation at the peripheral region (Fig. 3), leading to a high deposition of macromolecular proteins when comparing with the pure HSA. According to the classic fracture theory, a crack in a thin plate can be activated only when the suffered stress is larger than the critical stress for cracking, which can be written as [10]:

(1) $$\sigma_{c}=\sqrt{\frac{G_{c}\Omega E^{\prime }}{h}}$$

where *Gc* is the mechanical energy release rate that is defined by considering the sum of free energy during crack development, *Ω* is the cracking number and *E′* is the plane strain elastic modulus of the deposit at the cracking moment, *h* is the height of the thin plate. The critical stress ($\sigma_{c}$) is inversely proportional to the height (*h*). In our system, the deposition height in the peripheral region of HSA + NaCl is larger than that of pure HSA. The critical stress for cracking in HSA + NaCl is therefore less than the pure HSA, which means that the HSA + NaCl is much easier to crack during drop drying, and thus leading to a significantly higher number and width of radial cracks in HSA + NaCl desiccation patterns, but a shorter length of radial cracks compared to pure HSA. On the other hand, the effect of NaCl on HSA + NaCl desiccation patterns has also been found in the mixture of BSA + salt [21]. These authors indicated that the intermolecular interaction between BSA would be weakened by the existing salt, thus influencing the cracking patterns [21].

We then try to understand the formation of crystal patterns in the central region. During drop drying, the concentration of HSA gradually increases, as a result of continuous loss of water once it reaches to a critical value, HSA tends to aggregate and interact with each other to form a glassy film. This process is termed as gelation. Owing to the “coffee ring” effect [30,31], the gelation starts in the peripheral region of the sessile drop of HSA + NaCl, while the central region is still in a liquid state. It has been reported that the diffusion coefficient of the deposited macromolecular proteins in the gelled peripheral region approaches zero. By contrast, the diffusion coefficient of the dissolved inorganic salts in the liquid central region increases with their concentration [19]. The difference in the diffusion coefficient has been considered to cause the phase separation between gelled macromolecular proteins and dissolved inorganic salts, which could lead to the aggregation of inorganic salts in the liquid central region [19]. This is consistent with the explanation proposed by Pal et al. for their similar morphological observation in desiccation patterns of the mixture of Lys and PBS [Hierarchical exploration of drying patterns formed in drops containing lysozyme, PBS, and liquid crystals. Processes]. Unfortunately, we are still lacking of the experimental data for directly supporting this explanation.

### 3.4 Possible disease indicators of characteristic patterns

**Fig. 4:**
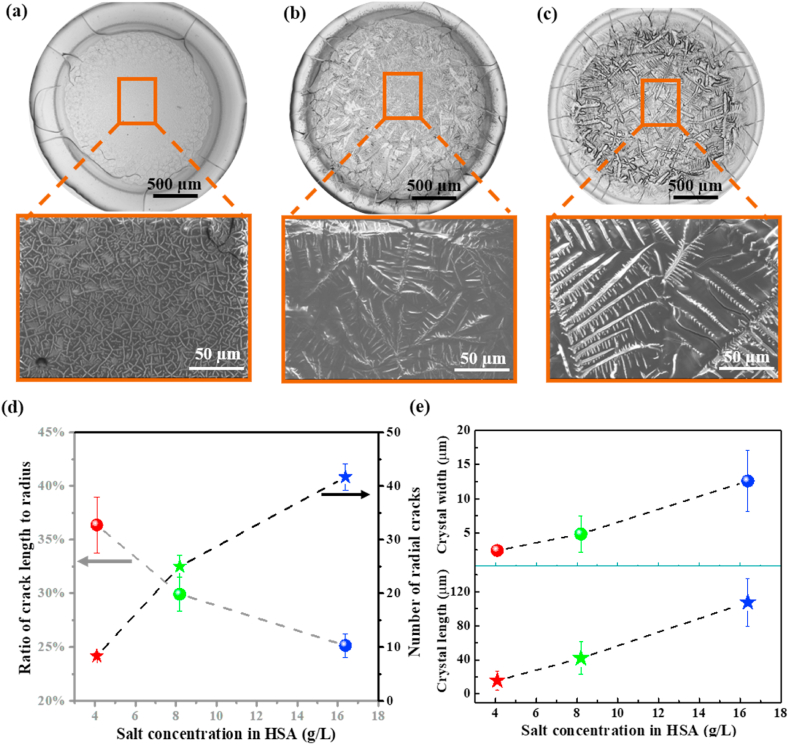
Desiccation patterns of HSA with different NaCl concentrations: (a) the desiccation patterns of mixture of 4.09 g/L NaCl and 50 g/L HSA and SEM image of the central region; (b) the desiccation patterns of mixture of 8.19 g/L NaCl and 50 g/L HSA and SEM image of the central region; (c) the desiccation patterns of mixture of 16.38 g/L NaCl and 50 g/L HSA and SEM image of the central region; (d) the ratio of crack length to radius and the number of cracks in desiccation patterns of HSA with different NaCl concentrations; (e) the crystal length and crystal width of the main grain of desiccation patterns of HSA with different NaCl concentrations.

Based on our mechanical understandings, we explored the correlation between characteristic patterns and different NaCl concentrations. We note that the NaCl concentration was selected according to the diseases of hyponatremia and hypernatremia in real biofluids. The resulting desiccation patterns are illustrated in Fig. 4(a–c). Herein, the mixture of 4.09 g/L NaCl and 50.00 g/L HSA, the mixture of 8.19 g/L NaCl and 50.00 g/L HSA and the mixture of 16.38 g/L NaCl and 50.00 g/L HSA are separately named as low concentration mixture, the normal concentration mixture and the high concentration mixture. All these desiccation patterns have two distinguished regions, i.e., peripheral region with radial cracks and central region with crystal patterns. However, there are obvious differences in morphological details of these desiccation patterns, as shown in Fig. 4(a–c).

We found that the number of radial cracks increased, while the length of radial cracks decreased with the increase of NaCl concentration. Further analysis by quantitative calculations revealed that the desiccation patterns of the low concentration mixture, the normal concentration mixture and the high concentration mixture exhibited 8 ± 0.5, 25 ± 2.0, and 42 ± 2.5 radial cracks, respectively, showing a remarkable increase in the number. Meanwhile, the normalized length of radial cracks was found calculated to ∼36%, ∼29%, and ∼25%, respectively (Fig. 4d), showing a substantial reduction with the increase of NaCl concentration. The difference in radial cracks is attributed to that a higher NaCl concentration in the HSA + NaCl causes an increase in the deposition height of desiccation patterns in the peripheral region, resulting in a lower critical stress required for cracking. Thus, it is more susceptible to cracking during drop drying, leading to a significant increase in the number of radial cracks in desiccation patterns of the high concentration mixture compared to the normal concentration mixture. Furthermore, the normalized length of radial cracks is shorter in the high concentration mixture than that of the normal concentration mixture and vice versa for low concentration mixtures.

For crystal patterns in the central region of the sessile drop, the distinct short pinstripe-shaped crystals are formed in the mixture with a low NaCl concentration of 4.09 g/L (Fig. 4a). When the NaCl concentration increases to a normal level of 8.19 g/L, crystal morphologies change to the dendritic shape (Fig. 4b). As the NaCl concentration further increases to a higher level of 16.38 g/L, the crystal scale, i.e., width and length, significantly increases (Fig. 4c). Our quantitative analysis indicates that, as the NaCl concentration increases, the average width of crystals increases from 2.4 μm to 4.8 μm, and finally to 12.6 μm; the average length also increases from 15.7 μm to 41.9 μm, and finally to 107.4 μm, as shown in Fig. 4e. We note that a higher concentration of NaCl would cause more NaCl aggregation in the central region, thus leading to varied crystal patterns.

## 4 Conclusion

In this work, the coupling effects of macromolecular protein (HSA) and inorganic salt (NaCl) on biological desiccation patterns have been disclosed. We have found that NaCl could enhance the “coffee ring” effect of HSA, resulting in radial cracking patterns with a larger width and an increase number. On the other hand, HSA experiences a gelation process that progresses from the peripheral region to the central region, leading to a spatial-temporal deviation in solidification degree that induces a higher concentration of NaCl in the central region; the crystal patterns therefore formed in the central region. The interplay between HSA and NaCl defines the final morphology of desiccation patterns, which were similar to plasma desiccation patterns. We have further correlated these characteristic patterns to the variation of the concentration of NaCl, which could be caused by diseases of hyponatremia and hypernatremia in real biofluids. These findings not only shed light on the understanding of biological desiccation patterns but also provide new avenues for diagnostic applications.

## Author contribution statement

Jihong Wang: Performed the experiments; Analyzed and interpreted the data; Contributed reagents, materials, analysis tools or data; Wrote the paper.

Min Zhang: Performed the experiments; Contributed reagents, materials, analysis tools or data; Wrote the paper.

Jun Wang: Analyzed and interpreted the data; Contributed reagents, materials, analysis tools or data; Wrote the paper.

Ruoyang Chen: Conceived and designed the experiments; Analyzed and interpreted the data; Contributed reagents, materials, analysis tools or data; Wrote the paper.

## Data availability statement

Data will be made available on request.

## Ethics declarations

Review and/or approval by an ethics committee was not needed for this study because all samples were biological/chemical reagents and whole experiments were performed without any ethical issues.

## Declaration of competing interest

The authors declare that they have no known competing financial interests or personal relationships that could have appeared to influence the work reported in this paper.
